# The Role of Preoperative ^18^F-fluorodeoxyglucose Positron Emission Tomography/Computed Tomography in Retroperitoneal Sarcoma

**DOI:** 10.3389/fonc.2022.868823

**Published:** 2022-05-30

**Authors:** Sung Jun Jo, Kyeong Deok Kim, So Hee Lim, Jinseob Kim, Seung Hyup Hyun, Jae Berm Park, Kyo Won Lee

**Affiliations:** ^1^Department of Surgery, Samsung Medical Center, Sungkyunkwan University School of Medicine, Seoul, South Korea; ^2^Department of Epidemiology, School of Public Health, Seoul National University, Seoul, South Korea; ^3^Department of Nuclear Medicine, Samsung Medical Center, Sungkyunkwan University School of Medicine, Seoul, South Korea

**Keywords:** PET, RPS, SUVmax, LPS, DDLPS

## Abstract

^18^F-fluorodeoxyglucose positron emission tomography/computed tomography (^18^F-FDG PET/CT) was used to predict pathologic grades based on the maximum standardized uptake value (SUVmax) in soft tissue sarcoma and bone sarcoma. In retroperitoneal sarcoma (RPS), the effectiveness of PET was not well known. This study was designed to investigate the association of SUVmax with histopathologic grade and evaluate the usefulness of ^18^F-FDG PET/CT before operation. Patients at Samsung Medical Center undergoing primary surgery for retroperitoneal sarcoma with preoperative ^18^F-FDG PET/CT imaging between January 2001 and February 2020 were investigated. The relationship between SUVmax and histologic features was assessed. The association of SUVmax with overall survival (OS), local recurrence (LR), and distant metastasis (DM) were studied. Of the total 129 patients, the most common histologic subtypes were liposarcoma (LPS; 68.2%) and leiomyosarcoma (LMS; 15.5%). The median SUVmax was 4.5 (range, 1- 29). Moreover, SUVmax was correlated with tumor grade (p < 0.001, Spearman coefficient; 0.627) and mitosis (p < 0.001, Spearman coefficient; 0.564) and showed a higher value in LMS (12.04 ± 6.73) than in dedifferentiated liposarcoma (DDLPS; 6.32 ± 4.97, p = 0.0054). SUVmax was correlated with pathologic parameters (tumor grade and mitosis) in RPS and was higher in the LMS group than the DDLPS group. The optimal SUVmax threshold to distinguish high tumor grade was 4.8. Those with a SUVmax greater than the threshold showed poor prognosis regarding OS, LR, and DM (p < 0.001).

## Introduction

Retroperitoneal sarcoma (RPS) is a rare neoplasm of mesenchymal origin derived from connective tissue. The most common histologic types are liposarcoma (LPS) and leiomyosarcoma (LMS), which account for 70% of all RPSs (1, 2).

Researches on the optimal treatment of RPS are in progress. Hospitals in many countries around the world are conducting continuous research together (2–4). For example, Almond, L.M., et al. was reported that neoadjuvant chemotherapy can improve the likelihood of negative resection margins in patients with locally advanced and high-risk primary sarcomas (5). Bonvalot, S., et al. was reported that preoperative radiotherapy had no clinical benefit on RPS (6). However, peri-operative treatments on RPS are still controversial and surgical resection, including that of adjacent organs, is accepted as the standard treatment (7–9).

Preoperative diagnosis and identification of tumor extent are important to determine extensive surgical resection including adjacent organs. Percutaneous biopsy and computed tomography (CT) are robust preoperative diagnosis methods and can safely determine histologic subtype and presence of metastasis (10, 11). However, percutaneous biopsy has limitations in that the accuracy is low (67.2%) and it is difficult to distinguish the tumor grade (12). In addition, CT scan has the disadvantage of being inaccurate in discriminating histologic subtypes of heterogenous tumors (13). Due to these limitations, diagnostic tool that can increase the accuracy of diagnosis is needed. The ^18^F-fluorodeoxyglucose positron emission tomography/computed tomography (^18^F-FDG PET/CT) can play a complementary role as it differentiate high-grade portion of heterogenous tumors and perform targeted biopsy (14).

There have been several studies on the use of ^18^F-FDG PET/CT in sarcomas, but most included both bone sarcoma and soft tissue sarcoma (15, 16). Alternatively, whole soft tissue sarcomas not specific to RPS have also been targeted (17, 18). Previously, our research team conducted a study on the association between maximum standardized uptake value (SUVmax) and retroperitoneal LPS (19). However, there was a limitation that only LPS was included.

In this study, we aimed to investigate the prognostic significance of SUVmax in RPS and to find out whether SUVmax shows different values depending on the histologic subtypes.

## Methods

### Patients

We retrospectively investigated patients undergoing primary surgery for RPS with preoperative ^18^F-FDG PET/CT imaging at Samsung Medical Center between January 2001 and February 2020. The diagnoses were determined according to the World Health Organization 2013 classification of specimens collected during surgery by pathologists specialized in sarcoma. The following patients were excluded: pediatric patients (those under 19 years of age); patients diagnosed with another malignant disease; patients who received pre-operative treatment, such as chemo-radiation therapy, before obtaining PET imaging; patients diagnosed with distant metastasis; and patients diagnosed with visceral sarcoma (tumors that clearly originated from a visceral organ, such as uterine sarcoma and sarcoma of the prostate, bladder, or vesicles), a benign tumor, carcinosarcoma, or a gastrointestinal tumor.

Data on underlying diseases, gender, BMI, and surveillance, such as [overall survival (OS), local recurrence (LR), and distant metastasis (DM)] were collected from patient medical records.

### Pathologic Characteristics

All pathologic records, based on surgical specimens, were reviewed by specialized sarcoma pathologists. Tumor histologic subtype, size, mitosis, necrosis, and multifocality were analyzed. Tumor grade was determined using the French Federation of Cancer Centers Sarcoma Group Grading System (FNCLCC).

### ^18^F-FDG PET/CT Imaging

All ^18^F-FDG PET/CT images were taken to confirm metastasis to other organs before surgery, and interpretations were made by nuclear medicine specialists. All patients fasted for at least 6 hours before PET/CT imaging, and their blood glucose level was required to be less than 200 mg/dL. Whole-body PET and unenhanced CT images were acquired using a PET/CT scanner (Discovery STE, GE Healthcare, Waukesha, WI, USA). Whole-body CT was performed using a 16-slice helical CT with 30 to 170 mAs adjusted to the patient’s body weight at 140-kVp and 3.75-mm section width. After the CT scan, an emission scan was performed from the thigh to the basal skull for 2.5 min per frame in three-dimensional mode 60 minutes after intravenous ^18^F-FDG injection (5.0 MBq/kg). The ordered subsets expectation-maximization algorithm (20 subsets and 2 iterations) with a 128  × 128 matrix and voxel size of 3.9 × 3.9 × 3.3 mm was used to reconstruct PET images utilizing CT data to correct attenuation. Regarding SUVmax measurement, we placed a spherical volume of interest with a diameter of 3 cm at a location where the tumor tissue had the highest metabolic activity using Volume Viewer (Advantage Workstation 4.4, GE Healthcare). SUVmax was normalized to patient body weight.

### Statistical Analysis

Factors affecting the prognosis of RPS were analyzed through univariate and multivariate Cox regression models. The Cox proportional hazards model was used to evaluate prognostic variables, and an estimated hazard ratio (HR) with its 95% confidence interval (95% CI) was presented. P < 0.05 was considered to represent a statistically significant comparison.

The Analysis of Variance (ANOVA) test was used to analyze the correlation between SUVmax and histologic subtypes. The receiver-operating characteristic (ROC) methodology was used to calculate the ideal threshold to distinguish high-grade sarcoma. The area under the curve (AUC) was calculated for each parameter using the non-parametric method to represent the overall predictive or prognostic performance.

Regarding survival analysis, Kaplan-Meier estimates and the log-rank test were used, and OS, LR, and DM were analyzed using time-to-event regression. Specifically, OS was calculated from the date of surgery to the date of death, LR was identified in CT scans, and the duration was calculated based on the CT scan date. DM was defined as a tumor found in organs such as liver, lung, brain, and bone, and the date of its diagnosis corresponded to when the tumor was detected by clinical symptoms or imaging tests. All analyses were performed using R version 4.0.4 (The R Core Team, Vienna, Austria).

### Ethical Approval

The study protocol conformed to the ethical guidelines of the Declaration of Helsinki and was approved by the Institutional Review Board of Samsung Medical Center (IRB No. 2021-09-062-001)

### Informed Consent

The need for informed consent was waived by the institutional review board of Samsung Medical Center due to the retrospective nature of the study.

## Results

### Clinicopathologic Data

In total, 136 patients who underwent primary surgery for RPS between 2001 and 2020 and underwent preoperative ^18^F-FDG PET/CT to determine the presence of metastasis were identified. Three patients with Ewing’s sarcoma were excluded. Four patients were excluded due to insufficient pathological data such as mitosis and necrosis. After excluding these patients, data from a total of 129 patients were investigated. The histologic subtypes were dominantly LPS (68.2%) and LMS (15.5%). DDLPS accounted for 68% of the LPS patients, followed by well-differentiated liposarcoma (WDLPS) and pleomorphic liposarcoma (PLS). There was no significant difference in the distribution of tumor grades. Demographic and clinicopathological details are shown in **Table 1**.

**Table 1:** Characteristics of patients.

Variable		Value
Age, years (mean)		56.4 ± 12.2
Gender (%)	F	67 (51.9)
	M	62 (48.1)
BMI, kg/m^2^ (mean)		23.5 ± 3.0
Underlying disease		
DM	Yes	11
	No	118
HTN	Yes	39
	No	90
Chronic renal disease	Yes	1
	No	128
Histologic subtype (%)	Well-differentiated liposarcoma	24 (18.6)
	Dedifferentiated liposarcoma	60 (46.5)
	Pleomorphic liposarcoma	4 (3.1)
	Leiomyosarcoma	20 (15.5)
	Malignant peripheral nerve sheath tumor	4 (3.1)
	Perivascular epithelioid cell tumor	1 (0.8)
	Other	16 (12.4)
FNCLCC grade (%)	1	29 (22.5)
	2	36 (27.9)
	3	64 (49.6)
SUVmax (median [range])		4.5 [0.4, 29.0]
Tumor size, mm (mean)		166.4 ± 101.3
Multifocality (%)	Yes	23 (17.8)
	No	106 (82.2)
Necrosis (%)	Absent	60 (46.5)
	<50%	60 (46.5)
	≥50%	9 (7.0)
Mitosis (%)	<9/10 HPF	95 (73.6)
	10-19/10 HPF	24 (18.6)
	≥20/10 HPF	10 (7.8)
Local recurrence (%)	Yes	54 (41.9)
	No	75 (58.1)
Distant metastasis (%)	Yes	17 (13.2)
	No	112 (86.8)
Follow up months after primary surgery, month (median[range])		37.8 [20.3, 71.9]

### Correlation Between SUVMmax and Pathologic Characteristics

**Figure 1:**
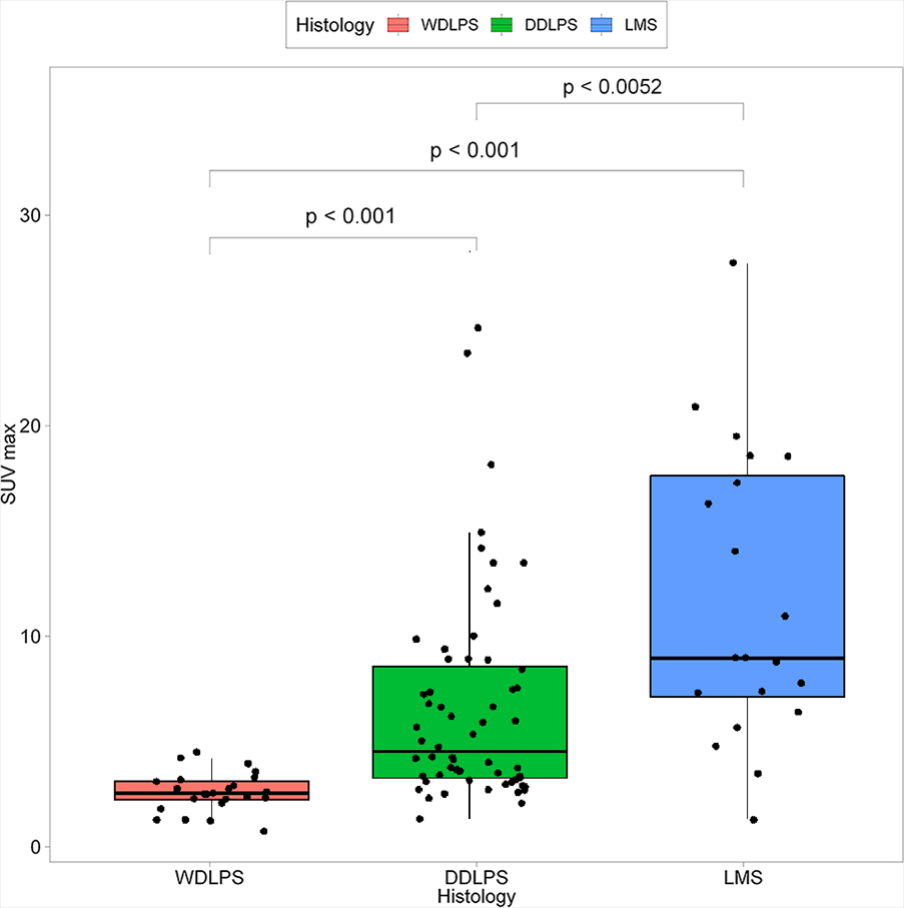
Comparison of median SUVmax with histologic subtypes.

The median SUVmax was 4.5 (range, 0.4-29). Tumor SUVmax was correlated with a higher tumor grade (p < 0.001, Spearman coefficient; 0.627) and mitosis (p < 0.001 Spearman coefficient; 0.564). In addition, SUVmax was different depending on the histologic subtype. The LPS group showed a lower SUVmax than the LMS group. When comparing SUVmax among the three groups, values were obtained in this order: WDLPS (2.32 ± 0.89), DDLPS (6.32 ± 4.97), and LMS (12.04 ± 6.73). The differences were statistically significant (**Figure 1**).

### Prognostic Factors for RPS and SUVmax

Univariate analysis of the prognostic factors associated with OS was performed considering all patients with RPS. The factors significantly associated with OS were high-tumor grade (grade III, p = 0.003), SUVmax (p < 0.001), mitosis (≥ 20/10 high power fields (HPF), p < 0.001), and necrosis (≥50%, p < 0.001). In the multivariate analysis, SUVmax (p = 0.004) was the only factor significantly associated with OS. When analyzing the OS factors by histologic subtype, tumor grade (grade III, p = 0.011) and SUVmax (p < 0.001) were significant prognostic factors in the LPS group, consistent with RPS. However, there were no statistically significant risk factors in the LMS group. The details of the analyses are shown in **Table 2**.

**Table 2:** Univariate and multivariate analyses of risk factors associated with overall survival.

Variables	Univariate		Multivariate	
	HR (95% CI)	p value	HR (95% CI)	p value
Male	1.9 (0.98,3.66)	0.057		
Age	1.03 (1,1.06)	0.033		
SUVmax	1.11 (1.07,1.16)	< 0.001	1.09 (1.03,1.15)	0.004
Tumor size	1 (1,1)	0.815		
FNCLCC grade: ref. = 1				
2	0.93 (0.19,4.61)	0.926	0.76 (0.15,4.01)	0.749
3	6.06 (1.84,19.98)	0.003	4.4 (0.83,23.45)	0.083
Histology: ref.=DDLPS				
WDLPS	0.35 (0.1,1.21)	0.097		
LMS	0.89 (0.33,2.41)	0.815		
MPNST	1.48 (0.34,6.38)	0.597		
Other	1.18 (0.43,3.22)	0.746		
Necrosis: ref.= Absent				
<50%	3.26 (1.46,7.28)	0.004	0.81 (0.24,2.74)	0.74
≥50%	6.49 (2.1,20.02)	0.001	1.37 (0.33,5.73)	0.666
Mitosis: ref.= <9/10 HPF				
10-19/10 HPF	2.26 (1.06,4.81)	0.035	0.7 (0.26,1.9)	0.484
≥20/10 HPF	4.63 (1.83,11.7)	0.001	0.77 (0.21,2.81)	0.69

Univariate analysis of prognostic factors for LR was performed considering all RPS patients. The SUVmax (p < 0.001), high tumor grade (p < 0.001), mitosis (≥20/10 HPF, p = 0.024), WDLPS (p = 0.004), LMS (p = 0.011) and necrosis (≥ 50%, p < 0.001) were significantly associated with LR. Within the multivariate analysis, the only factors independently associated with LR were high tumor grade (p = 0.014), WDLPS (p = 0.035) and necrosis (≥ 50%, p = 0.005). However, in the analysis conducted within histologic subtypes, SUVmax (p < 0.001) and high tumor grade (p = 0.002) were the main factors for LPS LR (**Table 3**).

**Table 3:** Univariate and multivariate analyses of risk factors associated with local recurrence.

Variables	Univariate		Multivariate	
	HR (95% CI)	p value	HR (95% CI)	p value
Male	1.14 (0.67,1.95)	0.632		
Age	1.01 (0.99,1.03)	0.503		
SUVmax	1.08 (1.04,1.12)	< 0.001	1.01 (0.94,1.08)	0.855
Tumor size	1 (1,1)	0.582		
FNCLCC grade: ref. = 1				
2	8.43 (1.9,37.39)	0.005	7.27 (1.57,33.75)	0.011
3	15.38 (3.69,64.04)	< 0.001	8.13 (1.54,42.92)	0.014
Histology: ref.=DDLPS				
WDLPS	0.12 (0.03,0.51)	0.004	0.19 (0.04,0.89)	0.035
LMS	2.32 (1.22,4.43)	0.011	1.93 (0.81,4.6)	0.137
MPNST	3.22 (0.96,10.78)	0.058	2.77 (0.75,10.19)	0.126
Other	0.46 (0.14,1.52)	0.206		
Necrosis: ref.= Absent				
<50%	3.35 (1.77,6.33)	< 0.001	1.56 (0.76,3.21)	0.23
≥50%	13.9 (5,38.6)	< 0.001	6 (1.74,20.7)	0.005
Mitosis: ref.= <9/10 HPF				
10-19/10 HPF	3.38 (1.79,6.39)	< 0.001	1.37 (0.61,3.09)	0.449
≥20/10 HPF	2.99 (1.15,7.75)	0.024	1.79 (0.4,7.91)	0.444

### Optimal Threshold to Distinguish High Grade Sarcoma

**Figure 2:**
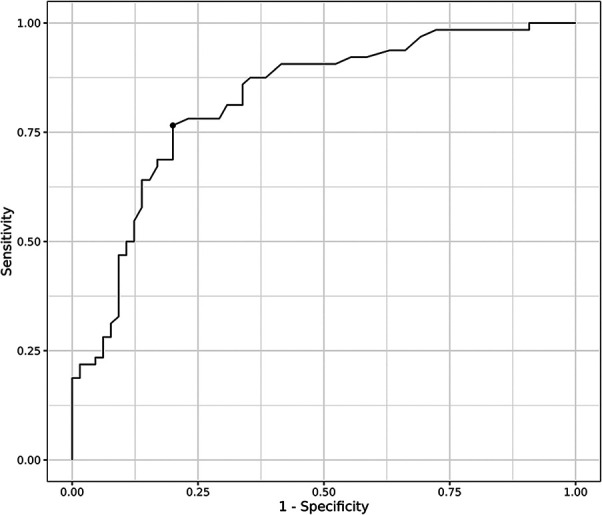
Receiver Operation Characteristic (ROC) curve for SUVmax.

Receiver Operating Characteristic (ROC) curve analysis demonstrated that the Area Under the ROC curve (AUC) for high tumor grade (Grade III) was maximal when the threshold SUVmax was 4.8. The AUC for high tumor grade at the cut-off SUVmax was 0.820 (p < 0.001). At this threshold, the values ​​of sensitivity and specificity were 0.77 and 0.80, respectively (**Figure 2**).

### Outcome prediction Using an Optimal SUVmax threshold

**Figure 3:**
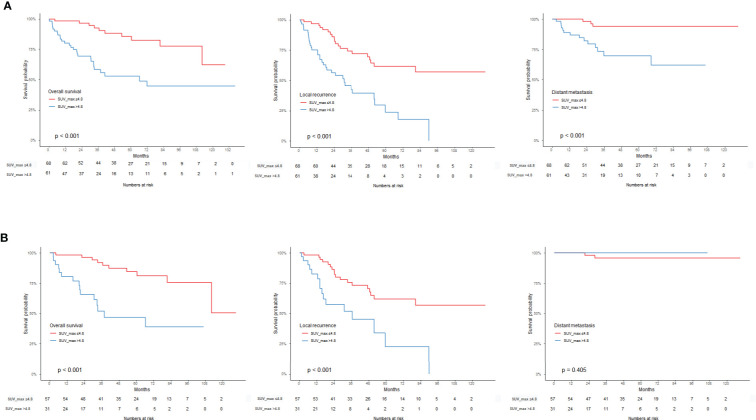
Kaplan-Meier survival graph compared to the SUVmax threshold of 4.8. **(A)** OS, LR, DM in RPS group, **(B)** OS, LR, DM in LPS group.

The SUVmax threshold was used to distinguish a high SUVmax group and a low SUVmax group survival analysis was performed with respect to OS, LR, and DM. Considering the entire RPS group, the high SUVmax group showed a poor prognosis regarding OS, LR, and DM (p < 0.001). When analyzed by histologic subtype, the LPS patients with high SUVmax showed poor prognosis regarding OS (p < 0.001) and LR (p = 0.004). However, there were no such differences in the LMS group (**Figure 3**).

## Discussion

This study analyzed the relationship between SUVmax and the pathologic characteristics and prognosis of RPS. We showed that SUVmax is associated with high-grade RPS. In addition, we demonstrated that the range of SUVmax varies according to histologic subtype.

### Distinction Between DDLPS and LMS

Our key finding was that higher SUVmax was found in LMS (12.04 ± 6.73) than DDLPS (6.32 ± 4.97). DDLPS and LMS are potential candidates for neoadjuvant chemotherapy, as the micro-metastasis potential is lowered, and unresectable tumors can be reduced in size before surgery (5). Anthracycline-based adjuvant chemotherapy is the cornerstone of first-line treatment for localized soft tissue sarcoma (20). However, based on many retrospective studies, different histology-driven -chemotherapy options can be applied to DDLPS and LMS. In addition, multi-center prospective research (STRASS-2) is ongoing to determine whether these treatments affect prognosis (21). The distinction between high-grade LPS and LMS is becoming increasingly important to clinical decision-making considering these studies. Our findings suggest that ^18^F-FDG PET/CT can be useful in distinguishing these two histologic subtypes preoperatively.

### Detecting High-Grade RPS Through ^18^F-FDG PET/CT Imaging

Due to its multifocal nature and large size, RPS can be difficult to target accurately during biopsy at the time of detection. In addition, preoperative biopsies tend to underestimate the final grade, most likely due to sampling error (22). For example, in LPS, when a solid portion and a fatty portion exist together, the high-grade portion is likely to be the solid portion. However, when there are several solid portions, it is difficult to predict the high-grade portion with CT. Because of these difficulties, the TARPSWG guidelines suggest that ^18^F-FDG PET/CT be available for defining biopsy target areas (2). The current study demonstrated that tumor -SUVmax was correlated with higher tumor grade (p < 0.001, Spearman coefficient; 0.627) and mitosis (p < 0.001 Spearman coefficient; 0.564). This result is similar to other studies showing the association between pathologic characteristics and SUVmax (22, 23). These results support the TARPSWG guidelines recommendation to set SUVmax as the biopsy target area.

### Prognosis Prediction Using SUVmax

A previous study conducted by our research team demonstrated that a SUVmax cut-off of  4.5 stratified RPS tumor grades and prognosis. In this study, only LPS was used, and there was a limitation in that SUVmax was determined in a heterogeneous population including metastatic and recurrent tumors (19). Subramaniam et al. also reported that when the SUVmax was higher than 5.0, the prognosis was poor, and high SUVmax and tumor grade were related. This study investigated a homogenous population; only the DDLPS and LMS groups were studied. However, the small number of patients has been mentioned as a limitation (22). In both studies referenced above, OS and relapse-free survival (RFS) were mentioned in the analysis of SUVmax and prognosis.

The current study investigated a relatively large number of patients given the low prevalence of RPS, excluding those with metastatic or recurrent tumors. In addition, the present study showed a correlation between SUVmax and DM, which has not been shown in other studies to our knowledge. The cut-off SUVmax (4.8) was a good measure for predicting prognosis but showed relatively low sensitivity (0.77) for predicting tumor grade and was not particularly useful in the LMS group. Therefore, our results indicate that ^18^F-FDG PET/CT may be a useful measure of prognosis or high tumor grade for LPS considering its relatively high specificity (0.8).

### Limitations

The current study is limited by its retrospective nature and the small number of LMS patients. A large-volume study is needed to find the SUVmax that can differentiate between DDLPS and LMS and to further evaluate the role of ^18^F-FDG PET/CT in recurrent and metastatic tumors.

## Conclusion

Tumor SUVmax was correlated with RPS pathologic parameters (tumor grade and, mitosis) and was higher in LMS than DDLPS. In addition, prognosis with respect to (OS, LR, and DM) was poor for patients with high SUVmax (p < 0.001). A SUVmax of 4.8 is the optimal threshold to rule out high-grade tumors, and prognosis can be predicted using this value.

## Data Availability Statement

The raw data supporting the conclusions of this article will be made available by the authors, without undue reservation.

## Ethics Statement

The studies involving human participants were reviewed and approved by Institutional Review Board of Samsung Medical Center. Written informed consent for participation was not required for this study in accordance with the national legislation and the institutional requirements.

## Author Contributions

SJ: Investigation, methodology, writing -original draft, writing – review & editing. KK: Investigation, resources. SL: Data curation, investigation. JK: Data curation, validation, visualization. SH: Writing – review & editing, investigation. JP: Project administration, resources. KL: Investigation, methodology, writing – review & editing. All authors contributed to the article and approved the submitted version.

## Funding

This research was supported by the SungKyunKwan University and the BK21 FOUR (Graduate School Innovation) funded by the Ministry of Education (MOE, Korea) and National Research Foundation of Korea (NRF).

## Conflict of Interest

The authors declare that the research was conducted in the absence of any commercial or financial relationships that could be construed as a potential conflict of interest.

## Publisher’s Note

All claims expressed in this article are solely those of the authors and do not necessarily represent those of their affiliated organizations, or those of the publisher, the editors and the reviewers. Any product that may be evaluated in this article, or claim that may be made by its manufacturer, is not guaranteed or endorsed by the publisher.
